# Sucralose Promotes Benzo(a)Pyrene-Induced Renal Toxicity in Mice by Regulating P-glycoprotein

**DOI:** 10.3390/antiox14040474

**Published:** 2025-04-16

**Authors:** Jun Hu, Ji Feng, Yan Bai, Zhi-Sheng Yao, Xiao-Yu Wu, Xin-Yu Hong, Guo-Dong Lu, Kun Xue

**Affiliations:** 1 School of Public Health, Fudan University, Shanghai 200032, China; 22211020134@m.fudan.edu.cn (J.H.); feng_ji@fudan.edu.cn (J.F.); 23211020112@m.fudan.edu.cn (Y.B.); 24111020094@m.fudan.edu.cn (Z.-S.Y.); 24211020227@m.fudan.edu.cn (X.-Y.W.); 2 Shanghai Municipal Center for Disease Control and Prevention, Shanghai 200336, China; hongxinyu@scdc.sh.cn

**Keywords:** sucralose, P-glycoprotein (PGP/ABCB1), benzo(a)pyrene, oxidative stress, nephrotoxicity

## Abstract

Background: Sucralose and benzo(a)pyrene (B[*a*]P) are widespread foodborne substances known to harm human health. However, the effects of their combined exposure on kidney function remain unclear. This study aimed to investigate the mechanisms by which sucralose and B[*a*]P induce kidney injury through P-glycoprotein (PGP/ABCB1), a crucial protein involved in cellular detoxification. Methods: C57BL/6N mice were co-treated with sucralose and B[*a*]P for 90 days to evaluate their impact on kidney histopathology and function. In vitro experiments assessed cell viability, reactive oxygen species (ROS) levels, and B[*a*]P accumulation by flow cytometry. Molecular docking and cellular thermal shift assay (CETSA) were used to determine the binding affinity of sucralose to PGP. Furthermore, PCR, Western blotting, and immunohistochemistry were performed to analyze the expression of PGP and its upstream transcription factors. Results: Ninety days of co-exposure to sucralose and B[*a*]P significantly exacerbated renal dysfunction in mice, as evidenced by the elevated level of serum creatinine and urea nitrogen, which could be reverted by ROS scavenger N-acetyl cysteine (NAC). In vitro, sucralose promoted cellular accumulation of B[*a*]P, consequently enhancing B[*a*]P-induced cell growth inhibition and ROS production. Consistently, B[*a*]P accumulation was enhanced by PGP knockdown in both HK2 and HEK-293 cells. Mechanistically, sucralose can directly bind to PGP, competitively inhibiting its efflux capacity and increasing intracellular B[*a*]P retention. Prolonged co-exposure further downregulated PGP expression, possibly through the reductions of its transcriptional regulators (*PXR*, *NRF2*, and *NF-κB*). Conclusions: Co-exposure to sucralose and B[*a*]P exacerbates renal injury by impairing PGP function. Mechanistically, sucralose inhibits PGP activity, resulting in the accumulation of B[*a*]P within renal cells. This accumulation triggers oxidative stress and inhibits cell growth, which demonstrates that sucralose potentiates B[*a*]P-induced nephrotoxicity by directly inhibiting PGP-mediated detoxification pathways, thus underscoring the critical need to evaluate toxicity risks associated with combined exposure to these compounds.

## 1. Introduction

Non-nutritive artificial sweeteners, widely adopted as calorie-free sugar alternatives, were ubiquitously incorporated into beverages, dairy products, and processed food formulations to meet consumer demand for reduced caloric intake [1]. Notably, the global market for artificial sweeteners expanded rapidly from USD 84 billion in 2014 to USD 1.11 trillion by 2020 [2]. Among non-nutritive artificial sweeteners, aspartame remains the most extensively utilized (18,500 metric tons annually), followed by saccharin (9700 metric tons), acesulfame potassium (6800 metric tons), and sucralose (3300 metric tons) [3].

Despite the establishment of Acceptable Daily Intake (ADI) standards for commonly used sweeteners by the Joint FAO/WHO Expert Committee on Food Additives (JECFA), a recent surveillance study reveals widespread non-compliance, including off-label applications, excessive dosing, and misuse [4,5]. The detection of artificial sweeteners and their metabolites in groundwater across multiple nations has raised concerns regarding their environmental persistence and potential toxicological impacts [3]. Of particular concern is sucralose because of its high metabolic stability. Pharmacokinetic studies indicate that only 10–20% of ingested sucralose is excreted renally, with merely 2% existing as glucuronide conjugates, suggesting its prolonged systemic retention and bioaccumulation risks [6].

Benzo[a]pyrene (B[*a*]P), a representative polycyclic aromatic hydrocarbon (PAH), is commonly found in the environment and generated through routine human activities [7]. It is primarily produced during the incomplete combustion of organic matter. Human exposure to B[*a*]P is almost unavoidable; it can occur through the ingestion of grilled foods, contaminated water, and inhalation of cigarette smoke [8]. The accumulation of B[*a*]P in the body can lead to significant toxicological effects, including DNA damage [9] and carcinogenesis [10]. Additionally, B[*a*]P exposure has been shown to increase serum urea and creatinine levels, as well as induce severe histopathological changes in the kidney [11]. Although both sucralose and B[*a*]P are commonly encountered in daily life, research on their combined exposure and potential synergistic effects remains limited.

P-glycoprotein (PGP) is an ATP-dependent transmembrane transporter predominantly expressed in excretory organs, including the kidney. It plays a critical role in the active efflux of intracellular xenobiotics, including environmental toxins and chemotherapeutic agents, thereby contributing to the maintenance of cellular homeostasis [12,13,14]. Dysfunction of PGP can lead to the abnormal accumulation of B[*a*]P in human breast cancer cells [15]. A recent study revealed that sucralose may disrupt xenobiotic metabolism by interfering with cellular drug efflux systems [16]. However, to date, there have been no reported studies investigating the combined effects of benzo[a]pyrene (B[*a*]P) and sucralose on normal tissues. The potential synergistic toxicity of these two compounds, particularly in the context of renal health, remains largely unexplored.

Therefore, this study aims to fill this gap by evaluating the combined effects of sucralose and B[*a*]P on kidney function. We thus conducted a 90-day subchronic exposure experiment in mice, accompanied by in vitro cellular experiments, to investigate the renal toxicity and underlying mechanisms associated with co-exposure to these substances. Furthermore, we aim to examine how sucralose influences the toxicological effects of B[*a*]P, particularly in identifying potential molecular pathways involved in their combined impact on kidney health.

## 2. Materials and Methods 

### 2.1. Animal Experiments

Seventy male C57BL/6N mice (20.1 ± 1 g) were purchased from Viton Lever (Beijing, China). Animals were housed in cages under controlled temperature (23 ± 1 °C) and relative humidity (50–55%) in a light/dark (12 h/12 h) cycle with ad libitum access to food and drinking water. All experiments were ethically approved by the Department of Laboratory Animal Science, Fudan University (approval number: 202409018S) and performed in accordance with the Association for Assessment and Accreditation of Laboratory Animal Care guidelines. After one week of acclimatization, the mice were randomly divided into seven groups (*n* = 10 per group), including control group (corn oil), the low-concentration sucralose group (5 mg/kg sucralose), the high-concentration sucralose group (15 mg/kg sucralose), the B[*a*]P alone group (50 mg/kg B[*a*]P), the combination group of low sucralose and B[*a*]P (5 mg/kg sucralose + 50 mg/kg B[*a*]P), the combination group of high sucralose and B[*a*]P (15 mg/kg sucralose + 50 mg/kg B[*a*]P), and the combination group of high sucralose and B[*a*]P plus NAC (100 mg/kg NAC). B[*a*]P was dissolved in corn oil and given by gavage every other day for 3 months, as described in previous studies [17,18]. The B[*a*]P dosing regimen was established through the integration of published toxicological data and regulatory safety guidelines, where literature-derived murine toxicity models employing 50–100 mg/kg doses [19,20] were reconciled with human occupational exposure thresholds (2.0 mg/kg) [21] through the application of the U.S. EPA-mandated 100-fold uncertainty factor for interspecies extrapolation. In the present study, high and low doses of sucralose were calculated through body surface area conversion, strictly adhering to the ADI values established by the U.S. FDA and WHO/JECFA. Consistently, mice received sucralose via drinking water at concentrations of 0.72 mg/mL and 0.17 mg/mL in a previous study [22]. This dosing regimen ensured cumulative exposure over the 10-week experimental period closely approximated the human ADI guidelines. The amount of water consumed was recorded. Mice were sacrificed at the end of the experiment, then their blood and kidneys were collected. A portion of the kidneys was fixed in 4% paraformaldehyde for histopathological examination, and the remaining tissues were rinsed with saline and stored in liquid nitrogen until further use.

### 2.2. Antibodies, Chemicals and Reagents

PGP (#22336-1-AP), GAPDH (#81640-5-RR) and Alpha Tubulin (#11224-1-AP) were purchased from Proteintech; while *γ*-H2AX (#9718); Caspase-3 (#9661); Bax (#2772); Bcl-2(#D17C4) were bought from Cell Signaling Technology (Danvers, MA, USA). The IHC experiments were carried out as before [23]. Sucralose (#S107614), benzo(a)pyrene (#B110854), verapamil hydrochloride (#V111249), N-acetylcysteine (#A105420), and corn oil (#C116023) were purchased from Aladdin (Shanghai, China).

### 2.3. Cell Culture and Cell Viability Assay

The HEK-293 and HK2 from the National Collection of Authenticated Cell Cultures were cultured in Dulbecco’s Modified Eagle Medium (DMEM) or DMEM/F12 50/50, respectively, and supplemented with 10% FBS (Biovision, Milpitas, CA, USA), 1% penicillin/streptomycin. Cells were cultured in a saturated humidity incubator at 37 °C, 5% CO_2_. Cell viability was assessed using a propidium iodide (#P4170, Sigma-Aldrich, St. Louis, MI, USA) exclusion assay in a flow cytometer (CytoFLEX, Beckman, Brea, CA, USA), as previously described [23].

### 2.4. Biochemical Analysis

Serum was collected from mice by ophthalmoplegia, then centrifuged at 1000× *g* for 10 min. Serum creatinine (CRE) and urea nitrogen (BUN) were measured using Creatinine assay test kit (#D799853, Sangon Biotech, Shanghai, China) or urea nitrogen assay kit (#D799850, Sangon Biotech), respectively.

### 2.5. Histology Staining

Renal tissues were sliced into 4 μm-thick sections and stained with hematoxylin and eosin (H&E) or periodic acid-Schiff (PAS). Tissue damage was quantified in a blinded manner. Renal tissue damage was scored according to the percentage of damaged tubules: 0, no damage; 1, less than 25% damage; 2, 25–50% damage; 3, 50–75% damage; 4, more than 75% damage [24]. The criteria for renal tubular injury include detachment of renal tubular epithelial cells, atrophic flattening of renal proximal tubules, and crumpled and deformed glomeruli.

### 2.6. Cellular Oxidative Stress Assay

HEK-293 cells or HK2 cells were seeded in 6-well plates at a density of 3 × 10^5^ cells per well and cultured until reaching approximately 60% confluency. Subsequently, the cells were treated with sucralose alone, B[*a*]P alone, or the combination as indicated for 24 h and 72 h. The positive control group was conducted according to the manuals of the reactive oxygen species detection kit (#S0033S, Beyotime Biotechnology, Shanghai, China) and the mitochondrial superoxide detection kit (#S0061S, Beyotime Biotechnology). The contents of reactive oxygen species in the cells of each group were detected by an Inverted Fluorescence Microscope (IX81, OLYMPUS, Tokyo, Japan) and flow cytometry (CytoFLEX, BECKMAN COULTER, Brea, CA, USA).

### 2.7. B[a]P Accumulation and Efflux Assay

Intracellular accumulation or efflux of B[*a*]P was examined by flow cytometry. Briefly, HEK-293 or HK2 cells were seeded in clear 12-well plates at a density of 2.5 × 10^5^ cells per well. For B[*a*]P accumulation assay, cells were treated as indicated and collected every 2 h for quantitative analysis of intracellular B[*a*]P levels by flow cytometry. For B[*a*]P efflux assay, cells were initially exposed to 2 μM B[*a*]P for 12 h. After a gentle wash with a complete medium, the culture medium was carefully replaced with a fresh complete medium with or without sucralose (10 μM). Cells were then incubated under light-protected conditions. The intracellular B[*a*]P concentrations were finally quantified via flow cytometry at 2 h intervals.

### 2.8. Cell Transient Transfection (PGP siRNA)

For transient knockdown, siRNAs (Sangon Biotech) against negative control (NC-si) or PGP (PGP-si1, PGP-si2, PGP-si3) were given to cells with the aid of Lipofectamine RNAiMAX (Thermo Fisher, Waltham, MA, USA). The nucleotide sequence of si-RNAs is summarized in Table 1.

### 2.9. Molecular Docking

The cryo-electron microscopy structure of nanodisk reconstituted drug-free human PGP/ABCB1 was retrieved from the Protein Data Bank (PDB #7A65) [25]. Hydrogen atoms and Gasteiger charges were added according to the AutoDock Vina documentation (version 1.2.0) [26] using the prepare receptor script from the ADFR suite [27]. Reference 3D models of sucralose, verapamil, and quinidine were retrieved from the Pubchem database, downloaded in sdf format, converted to pdb format using OpenBabel (version 2.4.1), and converted to pdbqt files using Autodocking (version 1.5.7). The pdbqt files of receptors and ligandswere used for docking experiments with AutoDock Vina [26]. We used the Orientation of Proteins in Membranes (OPM) database [28] (model 7a65.pdb) to adjust the coordinates and dimensions of the docking frames in the transmembrane region of the receptor. Vina 1.2 was run with an exhaustive parameter of 32, and docking was simulated 50 times per experiment. For each ligand, the corresponding binding poses on the receptor were examined and checked for consistency with the experimental data. The best binding poses associated with Vina’s built-in scoring function were then selected for presentation in this manuscript. PGP and docked ligands were visualized with PyMOL (version 4.6.0).

### 2.10. Western Blotting Assay

Cells were collected and lysed on ice utilizing RIPA lysate. After sufficient lysis, the cells were centrifuged at 12,000× *g* for 10 min at 4 °C, and the proteins of each treatment group were quantified using the BCA Protein Concentration Assay Kit (#P0012, Beyotime Biotechnology). Proteins were separated by SDS-PAGE and transferred to PVDF membranes (#ISEQ00010, Millipore, Bedford, MA, USA). After sealing the non-specific sites with 5% skimmed milk, the membranes were incubated overnight at 4 °C with the corresponding primary antibodies, followed by 1–2 h incubation with horseradish peroxidase-coupled secondary antibodies. Color development was performed using chemiluminescent reagents, and the intensity of protein bands was quantified using ImageJ software (version 1.52a) (NIH, Bethesda, MD, USA).

### 2.11. RNA Extraction, cDNA Synthesis, and RT-qPCR

Total RNA was extracted from treated cells by RNAiso Plus (#9019, Takara, Beijing, China) according to manufacturer’s instructions. cDNA synthesis was performed with the PrimeScript™ RT Master Mix (#RR036A, Takara), followed by qPCR analysis using gene-specific primers. Sequence-specific amplification was detected using TB Green^®^ Premix Ex Taq™ II FAST qPCR (#RR830A, Takara) on the CFX Opus Real-Time PCR System (Bio-Rad, Hercules, CA, USA). Primers were obtained from Sangon Biotech (Shanghai, China) and summarized in Table 2.

### 2.12. Cellular Thermal Shift Assay (CETSA)

The HK2 cell disruption solution was first prepared, divided into three groups by adding sucralose (10 μM), verapamil (10 μM) and PBS, and incubated at 37 °C for 30 min. The protein of each treatment group was then dispensed in 80 μL/tube into 10 PCR tubes and heated for 3 min at different temperatures (37 °C, 41 °C, 43 °C, 47 °C, 50 °C, 53 °C, 57 °C, 60 °C, 63 °C, 67 °C), and cooled at room temperature. Finally, the PGP protein expression levels were detected by Western blotting.

### 2.13. Statistical Analyses

Data were analyzed using GraphPad Prism software (version 9.5.0), and all data were expressed as mean ± standard deviation from at least three independent experiments. One-way ANOVA with Duncan’s test was used to assess significant differences between means, with statistical significance set at *p* < 0.05.

## 3. Results

### 3.1. Sucralose Enhanced B[a]P-Induced Subchronic Kidney Injury

**Figure 1:**
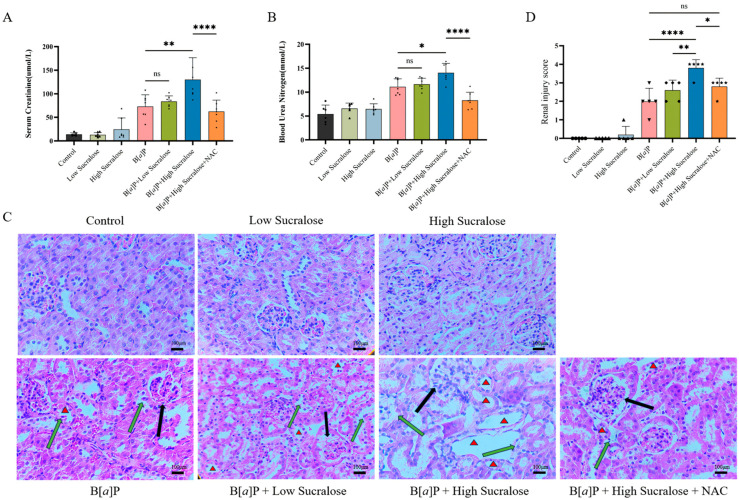
Subchronic treatment of sucralose enhanced B[*a*]P-induced kidney injury. Male C57BL/6N mice were treated with the indicated treatments for 90 days as in Material and Methods. The kidney injuries were monitored through quantification of (**A**) serum creatinine levels and (**B**) serum urea nitrogen levels or by (**C**) H&E histopathological examinations. Red triangles, atrophic flattening of renal proximal tubules. Black arrow, glomerular atrophy, and deformation. Green arrow: Renal tubular epithelial cell detachment. Scale bar: 100 µm. (**D**) Tubular injuries were scored (*n* = 5) and then summarized as described in Section 2.5. * *p* < 0.05; ** *p* < 0.01; **** *p* < 0.0001; ns, not significant.

To explore the effect of combined sucralose and B[*a*]P exposure on renal injury in mice, we administered B[*a*]P with or without sucralose treatment to mice over a period of 90 days. Renal damage was assessed by measuring blood creatinine and urea nitrogen levels and examining kidney tissue pathology. The experimental results showed that renal function abnormality was unobservable in all groups at 30 days of treatment (Figure S1A,B) and at 90 days of sucralose alone treatment. However, after 90 days of treatment, B[*a*]P treatment alone caused renal damage, as evidenced by elevated serum creatinine and blood urea nitrogen levels (Figure 1A,B) and significant pathological changes in tubular injury (Figure 1C,D). Notably, high-dose sucralose further exacerbated B[*a*]P-induced renal damage. In contrast, the ROS scavenger NAC attenuated the renal injury induced by the combined treatment (Figure 1A,D).

### 3.2. Sucralose Sensitized B[a]P-Induced Cell Growth Inhibition and ROS Production

**Figure 2:**
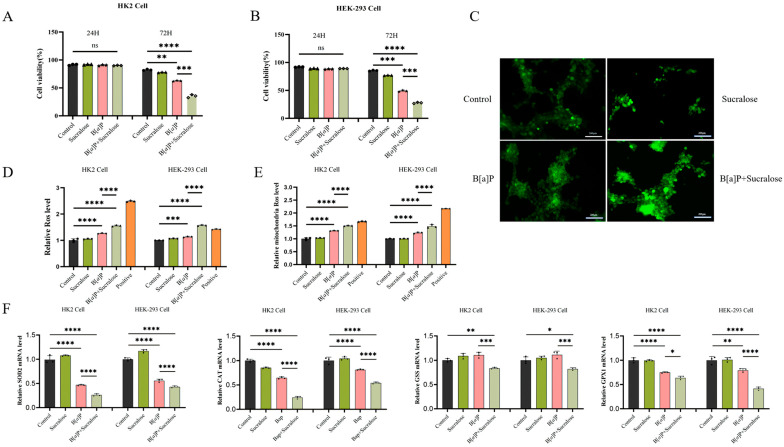
Sucralose could exacerbate B[*a*]P-induced cell growth inhibition and ROS production. HK2 (**A**) and HEK-293 cells (**B**) were exposed to sucralose (10 μM), B[*a*]P (20 μM), or combination for 24 or 72 h. The cell growth was then determined. (**C**) ROS levels in HK2 cells, as indicated by DCFDA fluorescence, were captured by fluorescent microscopy. Scale bar: 200 µm. (**D**) Total ROS levels in HK2 and HEK-293 cells after indicated treatments were determined by flow cytometry, and positive group refers to Rosup, a compound mixture that induces ROS. (**E**) Mitochondrial ROS levels in HK2 and HEK-293 cells with indicated treatments, positive group refers to mSoxUp, a compound mixture that induces superoxide formation in mitochondria. (**F**) Expression levels of *SOD2*, *CAT*, *GSS*, and *GPX1* genes in HK2 and HEK-293 cells were examined by qRT-PCR after indicated treatments. Each experiment was performed independently three times. * *p* < 0.05; ** *p* < 0.01; *** *p* < 0.001; **** *p* < 0.0001; ns, not significant.

To investigate the mechanisms underlying the enhanced renal injury by the combined treatment, we selected human renal tubular epithelial HK2 cells and human embryonic kidney HEK-293 cells for in vitro experiments. Our preliminary cytotoxicity assay indicated that sucralose, within the concentration range of 2.5–100 μM, exerted minimal effects on cell proliferation (Figure S2). Considering that the plasma concentration of sucralose in adults ranges from approximately 0.92 to 3.92 μM [29] and its known tendency to accumulate in renal tissues, we selected 10 μM sucralose for subsequent in vitro experiments. Likewise, B[*a*]P was used at 20 μM, a concentration commonly employed in in vitro studies [30,31,32]. Although there were no significant changes within 24 h, sucralose markedly sensitized B[*a*]P-induced cell growth inhibition in both HK2 and HEK-293 cells (Figure 2A,B). Since antioxidant NAC reduced renal injury induced by the combined treatment in mice (Figure 1), we examined the ROS generation by sucralose, B[*a*]P, and their combination in vitro. The results confirmed that sucralose could increase generations of both B[*a*]P-induced general ROS (Figure 2C,D) and mitochondrial-ROS levels (Figure 2E). Consistently, the combined treatment markedly decreased the expression of major antioxidant genes, including *SOD2*, *CAT*, *GSS*, and *GPX1* (Figure 2F). Taken together, these results suggest that sucralose could exacerbate B[*a*]P-induced cell growth inhibition and ROS production.

### 3.3. Sucralose Promotes B[a]P Intracellular Accumulation and Retention in Renal Cells

**Figure 3:**
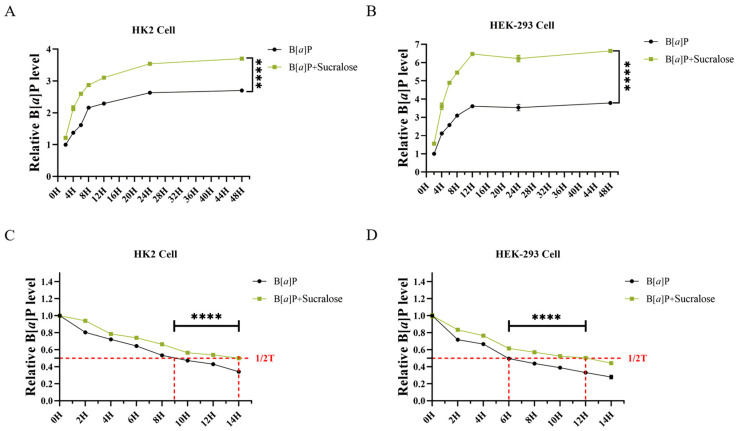
Sucralose enhanced the intracellular accumulation and retention of B[*a*]P. HK2 (**A**) and HEK-293 cells (**B**) were treated with B[*a*]P in the presence or absence of sucralose (10 μM). The intracellular B[*a*] concentration was then determined by flow cytometry in a time course, and the relative fluorescence values normalized to the 2 h fluorescence value of the B[*a*]P group (set as 100%). (**C**,**D**) HK2 (**C**) and HEK-293 cells (**D**) were treated with B[*a*]P alone for 12 h. After replacement with the sucralose-containing fresh medium, the retention of B[*a*]P was determined in 2 h-interval. Each experiment was performed independently three times. **** *p* < 0.0001.

Next, we tested whether sucralose influences the intracellular accumulation of B[*a*]P in vitro. The combined treatment significantly caused a higher level of B[*a*]P accumulation in both HK2 and HEK-293 cells, compared to B[*a*]P alone treatment (Figure 3A,B). Since cellular efflux contributes to B[*a*]P accumulation, we next assessed B[*a*]P efflux. After cells were treated with B[*a*]P alone for 12 h, the culture medium was replaced with sucralose, and then the intracellular level of B[*a*]P was determined at a 2 h interval. Notably, the addition of sucralose could reduce B[*a*]P efflux efficiency, as the median duration of B[*a*]P retention was increased from 9.48 ± 0.01 h to 13.55 ± 0.01 h in HK2 cells (Figure 3C) and 6.01 ± 0.11 h to 11.29 ± 0.02 h in HEK-293 cells (Figure 3D). Therefore, these findings implied that sucralose might inhibit B[*a*]P efflux in renal cells.

### 3.4. Knockdown of PGP Protein Further Promotes Sucralose-Induced B[a]P Accumulation

**Figure 4:**
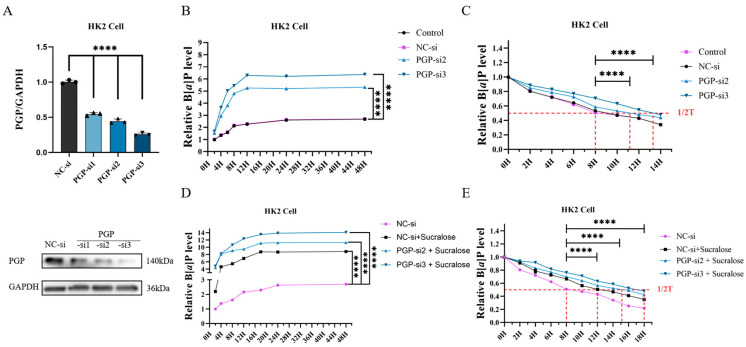
PGP plays a critical role in sucralose-induced accumulation of B[*a*]P. (**A**) HK2 cells were treated with PGP siRNA, and then the protein level of PGP was detected. (**B**,**C**) Effect of knockdown of PGP alone on B[*a*]P accumulation (**B**) and retention (**C**) in HK2 cells. (**D**,**E**) Effect of knockdown of PGP on sucralose-induced B[*a*]P accumulation (**D**) and retention (**E**) in HK2 cells. Each experiment was performed independently three times. **** *p* < 0.0001.

In order to examine whether sucralose-promoted B[*a*]P accumulation is related to cellular efflux function, we further determined the role of PGP (Figure 4A), a transmembrane protein capable of effluxing B[*a*]P. As expected, knockdown of PGP promoted B[*a*]P accumulation (Figure 4B) and reduced B[*a*]P efflux when compared with the NC-si treatment group (Figure 4C, as the median of retention increased from 8 h in the NC-si group to 11 in PGP-si2 or 13 h in PGP-si3 group). Moreover, PGP knockdown further enhanced sucralose-induced B[*a*]P accumulation (Figure 4D) and retention (Figure 4E, comparing 12 h in the NC-si-sucralose group to 15 or 18 h in the NGP-si + sucralose groups).

### 3.5. Sucralose Inhibits B[a]P Efflux by Binding Specifically to PGP

**Figure 5:**
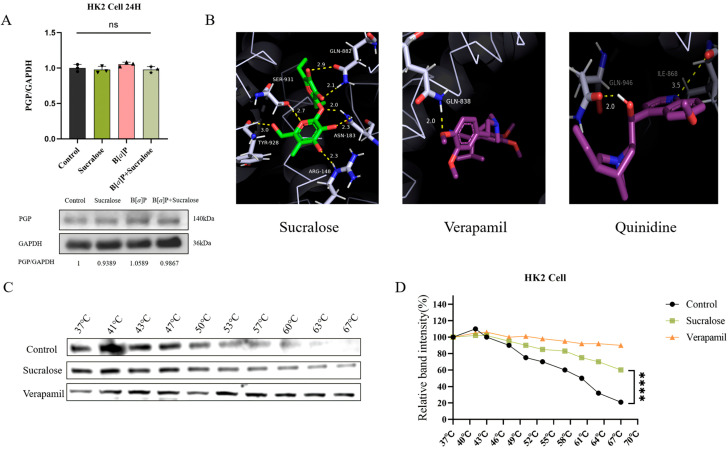
Sucralose directly binds to PGP. (**A**,**B**) Expression of PGP protein in HK2 cells after the indicated treatments for 24 h. (**B**) Optimal binding postures of sucralose to PGP, together with verapamil and quinidine as positive controls. (**C**,**D**) CETSA experiments were performed to confirm the interaction between PGP protein and sucralose or verapamil (**C**). The quantitative results were summarized in panel D. Each experiment was performed independently three times. **** *p* < 0.0001; ns, not significant.

To investigate whether sucralose directly acts on PGP protein, we first examined the effect of sucralose on PGP protein expression. After 24 h of treatment, none of sucralose alone, B[*a*]P alone, and the combined treatment affected PGP protein expression (Figure 5A). This result is inconsistent with the enhanced intracellular accumulation and retention of B[*a*]P by sucralose (Figure 3), prompting us to investigate whether sucralose can directly interfere with PGP activity.

We performed virtual docking experiments with sucralose in the PGP transmembrane structural domain. As positive controls, verapamil and quinidine, two known strong inhibitors of PGP, were used for the virtual docking experiments. A complete report of docking positions was provided in Table S1, along with calculated metrics, including binding affinity and root mean square deviation (RMSD). The molecular docking results showed that the docking energy of PGP with sucralose was the strongest at −7.35, even exceeding that of verapamil (−5.31) and quinidine (−5.51). The bindings posed with the highest binding energy, selected from 50 simulated dockings, were shown in Figure 5B, illustrating that sucralose binds more tightly to PGP.

To experimentally validate the in silico predicted binding interaction between sucralose and P-glycoprotein (PGP), we performed a CETSA assay. The results demonstrated that PGP was more stable and less prone to degradation after binding to sucralose (Figure 5C,D). Therefore, these results suggest that sucralose binds to PGP with more affinity, thus leading to the accumulation of B[*a*]P in renal cells.

### 3.6. Sucralose Combined with B[a]P Inhibits the Expression of PGP and Its Upstream Transcription Factors in the Long Run

**Figure 6:**
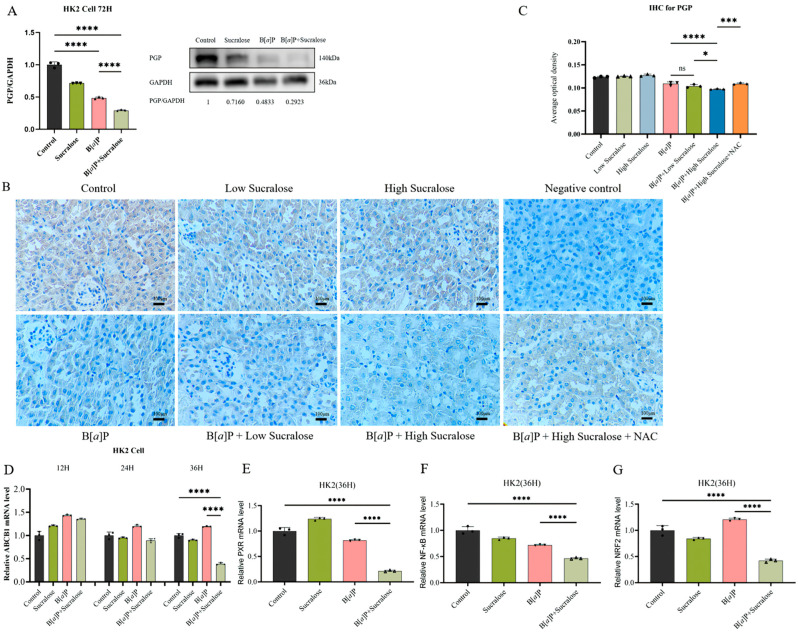
The combined treatment inhibited PGP transcription in the long run. (**A**) The expression of PGP protein was determined in HK2 after the indicated treatments for 72 h. (**B**,**C**) PGP expression in mouse kidney tissues was analyzed by IHC. Representative images (**B**) and quantitative analysis (**C**) were shown. (**D**) PGP (*ABCB1*) mRNA levels were determined by qRT-PCR in HK2 cells after 12 h, 24 h, and 36 h of different treatments. (**E**–**G**) Upstream transcription factors *PXR* (**E**), *NF-κB* (**F**), and *NRF2* (**G**) mRNA levels were determined in HK2 cells after 36 h of the indicted treatments. Each experiment was performed independently three times. * *p* < 0.05; *** *p* < 0.001; **** *p* < 0.0001, ns, not significant. Scale bar: 100 µm.

Although none of the treatments within 24 h affected PGP protein expression, after 72 h of treatment, both sucralose alone and B[*a*]P alone reduced PGP protein expression in HK2 cells. Moreover, the combined treatment resulted in a further decrease in PGP protein levels (Figure 6A). This observation was further confirmed in the treated mice kidney tissues, as the combined treatment decreased PGP protein expression when compared with B[*a*]P alone (Figure 6B,C).

In a parallel manner, the combined treatment also reduced PGP mRNA expression after 36 h incubation but not after 12 or 24 h (Figure 6D), suggesting that the combined treatment might modulate the transcriptional regulation of PGP. To investigate this further, we assessed the expression of upstream transcription factors of PGP, including *PXR*, *NRF2*, and *NF-κB* [33,34,35]. The results demonstrated that sucralose and B[*a*]P co-treatment reduced the mRNA expression of all three transcription factors (Figure 6E,G).

## 4. Discussion

Artificial sweeteners, introduced to the market as food ingredients in the 19th century [36], were once generally considered safe due to their metabolic inertness. Some studies have shown that long-term consumption of commonly used artificial sweeteners does not cause adverse metabolic effects [37]. However, with the classification of aspartame as a class 2B carcinogen, the safety of artificial sweeteners has triggered a global debate [38]. Although epidemiological data have not found solid evidence of the carcinogenic effects of sucralose [39], some studies have reported its adverse non-tumor effects. For instance, short-term consumption of carbohydrate-containing sucralose has been shown to impair human neuro- and metabolic sensitivity to sugar [40], while long-term sucralose intake has been associated with dysbiosis of the gut microbiota and alterations in glucose and insulin levels in healthy young adults [41].

B[*a*]P is a class I carcinogen known to damage multiple organs, including the liver, lungs, and kidneys. Its carcinogenic and toxic effects are largely due to its ability to form reactive metabolites that can bind to cellular DNA, leading to mutations and cellular dysfunction. B[*a*]P has been shown to induce ROS, DNA damage and apoptosis via its metabolite benzo[a]pyrene-7,8-dihydrodiol-9,10-epoxide (BPDE), in turn induces hepatic and renal injuries after prolonged exposure [19,42]. While B[*a*]P itself is highly harmful, its toxicity can be exacerbated when combined with other environmental pollutants, leading to more severe adverse effects on human health. Studies have demonstrated that simultaneous exposure to B[*a*]P and phthalates (PAEs), can increase the risk of developing pregnancy-related hypertension [43]. Dibutyl phthalate (DBP), another common phthalate ester, has been shown to exacerbate B[*a*]P-induced renal damage in rats [44]. These results highlight the critical importance of studying the combined exposure to environmental pollutants. Notably, the present study found that sucralose treatment could exacerbate B[*a*]P-induced renal injury in mice and inhibit cell growth in vitro (Figure 1). Although both sucralose and B[*a*]P are common environmental exposures, this study, to our knowledge, might be the first report to explore the combined effects of sucralose and B[*a*]P.

Maintaining oxidative homeostasis is crucial for cell survival, in which antioxidant enzymes may play an important role. It has been reported that B[*a*]P significantly increases ROS production in human hepatocellular carcinoma cells [36] and rat hepatocytes [45]. Among the antioxidant enzymes, superoxide dismutase (SOD) levels are inhibited upon exposure to 10 μM B[*a*]P [46]. Furthermore, B[*a*]P impairs antioxidant capacity by disrupting the activity of SOD3 and GPX4 proteins [45]. Concurred with the previous studies, we confirmed that the combined treatment of sucralose and B[*a*]P increases the ROS level in both HK2 and HEK-293 cells, as well as causes downregulation of several antioxidant genes, including *SOD2*, *CAT*, *GSS*, and *GPX*1 (Figure 2). The enhanced inhibitions of both cell growth and antioxidant enzymes by the combined treatment may indicate that sucralose may sensitize B[*a*]P-induced toxicities.

PGP, an ATP-dependent transmembrane transporter protein, is widely distributed in excretory organs, such as the kidney, liver, and intestines. It plays a crucial role in the excretion of exogenous toxins and drugs, thereby protecting cells from harmful substances, including PAHs [12,13]. Thus, PGP-mediated toxin efflux is regarded as a key component of the detoxification system in the body [47]. Consistent with these findings, we found that sucralose treatment, as well as knockdown of PGP, promotes B[*a*]P accumulation in renal cells (Figure 4). These results initially led us to question whether sucralose contributes to the accumulation of B[*a*]P in renal cells by affecting the expression level of PGP. However, the results of Western blotting showed that the combined treatment of sucralose and B[*a*]P for 24 h failed to alter PGP protein levels (Figure 5A), suggesting a possible direct action of sucralose on PGP activity. Molecular docking and CETSA assays confirmed that sucralose binds to PGP with high affinity (Figure 5), which is consistent with a prior report by Laura Danner et al. [16]. This direct interaction might contribute to the increased intracellular accumulation and retention of B[a]P, which in turn enhanced B[*a*]P-induced cytotoxicity and renal injury.

Interestingly, after long-term co-treatment (72 h) with both sucralose and B[*a*]P, we observed a significant decrease in PGP protein expression levels (Figure 6). Particularly, the combined treatment decreased PGP (*ABCB1*) mRNA expression after 36 h rather than after 12 or 24 h. This inconsistency can be explained by the relatively long protein half-life of PGP (~27 h) [48]. Further mechanistic analyses demonstrated that the combined treatment downregulated the upstream transcriptional regulators of PGP, including *NF-κB*, *NRF2*, and *PXR*, with *NRF2* exhibiting the most consistent pattern of suppression. Collectively, these data support a dual-phase modulation of PGP: (1) The direct binding activity of sucralose to PGP may enable its sensitization effects to enhance B[*a*]P intracellular accumulation and consequent cytotoxicity; (2) The prolonged sucralose treatment (more than 36 h) might impair PGP (*ABCB1*) transcription through the modulation of the upstream transcriptional factors. These two modulatory actions of sucralose against PGP may exacerbate B[*a*]P-induced toxicity. Whether sucralose can affect other xenobiotics through PGP inhibition and sensitize its toxicity warrants further investigation.

This study, however, has some limitations. First, this study primarily investigated the sub-chronic toxicity of sucralose and B[*a*]P, with a specific focus on the kidneys. Further research is needed to explore the effects of co-exposure to sucralose and B[*a*]P on other organs (e.g., liver, intestines, etc.) over a longer duration to provide a more comprehensive understanding of their biological effects. Second, although the present study has revealed that PGP plays a key role in sucralose-mediated accumulation of B[*a*]P, the precise molecular mechanism underlying PGP’s involvement, such as how sucralose and B[*a*]P regulate transcription of ABCB1, requires further investigation. Third, it is yet unclear whether co-exposure to sucralose and B[*a*]P can predispose to human disease. Prospective cohort studies might shed light on this essential question. Taken together, a deeper understanding of these mechanisms will allow for a more accurate analysis of the related biological processes and provide a stronger theoretical foundation for future studies and risk assessments.

## 5. Conclusions

In conclusion, the present study demonstrates that the combined treatment with sucralose and B[*a*]P exacerbates renal impairment in mice. Mechanistically, sucralose promotes the accumulation of B[*a*]P within cells by inhibiting the efflux function and expression of PGP. This accumulation, in turn, triggers oxidative stress and inhibits cell growth. Thus, sucralose may enhance the toxic effects of B[*a*]P by interfering with the detoxification protein PGP. These findings highlight the need for further investigations into the combined effects of sucralose and B[*a*]P, given their common exposure and significant public health implications.

## Figures and Tables

**Table 1:** Sequences of si-RNAs.

Name	Sense (5′ to 3′)
NC-si	UUCUCCGAACGUGUCACGU
PGP-si1	CAAUGUUUCGCUAUUCAAA
PGP-si2	GGAGGAGCAAAGAAGAAGA
PGP-si3	GGAAUGUUCUUUCAGUCAA

**Table 2:** Information of primers.

Genes	Accession Number	Forward Primer Sequence	Reverse Primer Sequence
*ABCB1*	NM_001348946	GTCCCAGGAGCCCATCCT	CCCGGCTGTTGTCTCCATA
*NRF2*	NM_006164	TCAGCGACGGAAAGAGTATGA	CCACTGGTTTCTGACTGGATGT
*NF-κB*	NM_003998	AACAGAGAGGATTTCGTTTCCG	TTTGACCTGAGGGTAAGACTTCT
*PXR*	NM_003889	AAGCCCAGTGTCAACGCAG	GGGTCTTCCGGGTGATCTC
*SOD2*	NM_001322816	GCTCCGGTTTTGGGGTATCTG	GCGTTGATGTGAGGTTCCAG
*CAT*	NM_001752	TGGAGCTGGTAACCCAGTAGG	CCTTTGCCTTGGAGTATTTGGTA
*GSS*	NM_000178	GGGAGCCTCTTGCAGGATAAA	GAATGGGGCATAGCTCACCAC
*GPX1*	NM_000581	CAGTCGGTGTATGCCTTCTCG	GAGGGACGCCACATTCTCG
*ACTB*	NM_001101	GGACTTCGAGCAAGAGATGG	AGCACTGTGTTGGCGTACAG

## Data Availability

All data mentioned in the manuscript can be provided by the corresponding author upon reasonable request.

## Supplementary Materials

The following supporting information can be downloaded at https://www.mdpi.com/article/10.3390/antiox14040474/s1, Figure S1: Sucralose combined with B[*a*]p induces subchronic kidney injury. (A) Blood creatinine levels in the indicated treatment groups. (B) Blood urea nitrogen levels in the indicated treatment groups. ns, not significant; Figure S2: Effect of sucralose on HK2 cell proliferation; Table S1: Detailed results of sucralose, verapamil and quinidine docking with PGP simulations.
