# Supplementary material for: Sucralose Promotes Benzo(a)Pyrene-Induced Renal Toxicity in Mice by Regulating P-glycoprotein
Source: Antioxidants (Basel). 2025 Apr 16;14(4):474. doi: 10.3390/antiox14040474 (PMC12024012; doi:10.3390/antiox14040474)
Supplement: Supplementary file 1 [file antioxidants-14-00474-s001.zip › antioxidants-3548758-supplementary.pdf]

Supplementary Material

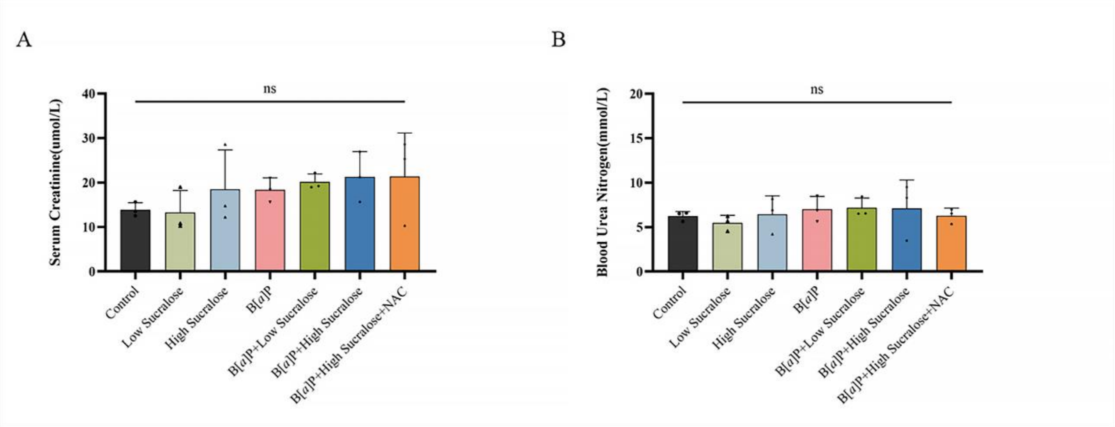

Figure S1. Sucralose combined with B[α]p induces subchronic kidney injury. (A) Blood creatinine levels in the indicated treatment groups. (B) Blood urea nitrogen levels in the indicated treatment groups. ns, not significant.

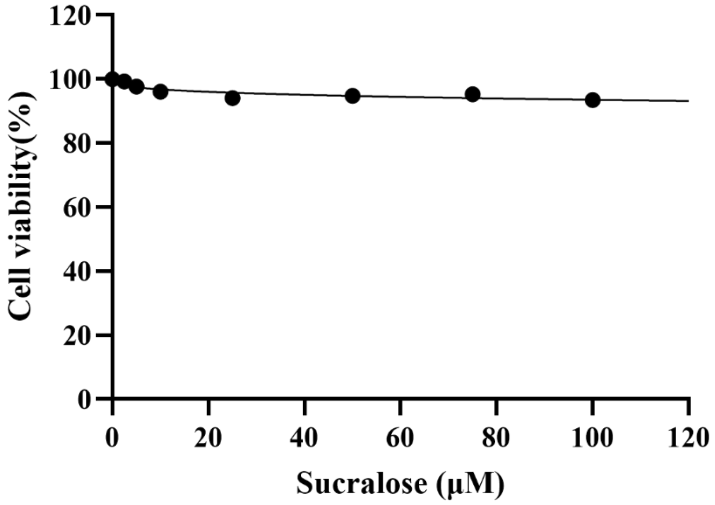

Figure S2. Effect of sucralose on HK2 cell proliferation.

Table S1: Detailed results of sucralose, verapamil and quinidine docking with PGP simulations.

		**Sucralose**				**Verapamil**	
Rank	Run	Binding Energy(kcal/mol)	RMSD	Rank	Run	Binding Energy(kcal/mol)	RMSD
1	14	-7.35	0.00	1	21	-5.62	0.00
2	17	-7.23	0.00	2	17	-5.61	0.00
3	23	-6.52	0.00	3	49	-5.50	0.00
3	29	-6.25	0.88	4	16	-5.09	0.00
4	46	-6.43	0.00	5	41	-5.08	0.00
4	32	-6.23	0.80	6	22	-5.03	0.00
5	15	-6.40	0.00	7	28	-4.93	0.00
6	25	-6.23	0.00	8	31	-4.85	0.00
7	9	-6.20	0.00	9	19	-4.80	0.00
8	2	-6.09	0.00	10	39	-4.76	0.00
9	7	-6.00	0.00	11	15	-4.72	0.00
9	48	-5.60	1.86	12	37	-4.63	0.00
10	1	-6.00	0.00	13	2	-4.62	0.00
11	49	-5.94	0.00	14	25	-4.57	0.00
11	50	-5.80	1.27	15	29	-4.44	0.00
12	26	-5.89	0.00	16	11	-4.02	0.00
13	27	-5.84	0.00	17	13	-3.94	0.00
14	8	-5.72	0.00	18	20	-3.90	0.00
15	28	-5.70	0.00	19	30	-3.81	0.00
16	34	-5.60	0.00	20	38	-3.73	0.00
17	42	-5.51	0.00	21	43	-3.71	0.00
17	35	-5.35	1.63	22	45	-3.70	0.00
18	6	-5.51	0.00	23	44	-3.68	0.00
18	5	-5.41	1.64	24	4	-3.64	0.00
19	13	-5.46	0.00	25	24	-3.58	0.00
20	36	-5.35	0.00	26	35	-3.53	0.00
21	10	-5.33	0.00	27	8	-3.46	0.00
22	21	-5.30	0.00	28	34	-3.09	0.00
23	12	-5.29	0.00	29	33	-3.07	0.00
24	38	-5.28	0.00	30	10	-3.05	0.00
25	37	-5.27	0.00	31	1	-3.02	0.00
26	4	-5.17	0.00	32	12	-2.92	0.00
27	39	-5.16	0.00	33	50	-2.78	0.00
27	30	-4.66	1.07	34	26	-2.77	0.00
28	43	-5.11	0.00	35	9	-2.76	0.00
28	22	-5.09	0.59	35	27	-1.68	1.98
29	40	-4.96	0.00	36	3	-2.75	0.00
30	3	-4.95	0.00	37	48	-2.74	0.00

31	44	-4.95	0.00	38	32	-2.56	1.78
32	20	-4.91	0.00	38	5	-2.42	0.00
33	33	-4.85	0.00	39	47	-2.54	0.00
33	11	-4.85	0.86	40	46	-2.50	0.00
34	41	-4.81	0.00	41	6	-2.46	0.00
35	24	-4.81	0.00	42	7	-2.38	0.00
36	18	-4.76	0.00	43	36	-2.33	0.00
37	45	-4.73	0.00	44	18	-2.17	0.00
38	19	-4.70	0.00	45	23	-2.03	0.00
39	31	-4.68	0.00	46	42	-1.75	0.00
40	47	-4.63	0.00	47	14	-1.72	0.00
41	16	-4.34	0.00	48	40	-1.71	0.00

		**Quinidine**	
Rank	Run	Binding Energy(kcal/mol)	RMSD
1	44	-5.51	0.00
1	40	-5.33	0.68
1	19	-5.30	1.87
1	45	-4.84	1.35
1	42	-4.57	1.91
2	24	-5.38	0.00
2	16	-5.19	0.96
2	10	-4.99	1.19
2	46	-4.64	1.40
2	43	-4.61	0.9
2	8	-4.43	1.01
3	29	-5.16	0.00
4	27	-5.05	0.00
5	2	-4.98	0.00
6	39	-4.98	0.00
6	37	-4.44	1.58
7	28	-4.96	0.00
7	17	-4.89	0.25
7	26	-4.87	0.59
7	13	-4.86	1.14
7	22	-4.82	1.19
8	33	-4.92	0.00
9	32	-4.86	0.00
9	48	-4.85	0.43
9	15	-4.83	0.34
9	20	-4.81	0.40
9	47	-4.80	0.53
9	38	-4.74	0.64
9	6	-4.54	1.40

9	49	-4.54	1.43
9	34	-4.52	1.41
9	5	-4.22	1.55
10	4	-4.79	0.00
11	30	-4.77	0.00
12	25	-4.75	0.00
13	3	-4.72	0.00
14	1	-4.72	0.00
15	12	-4.66	0.00
16	9	-4.65	0.00
16	11	-4.57	0.71
17	35	-4.54	0.00
18	36	-4.50	0.00
19	21	-4.47	0.00
20	14	-4.33	0.00
21	31	-4.31	0.00
22	50	-4.25	0.00
23	7	-4.12	0.00
24	18	-4.03	0.00
25	23	-4.00	0.00
26	41	-3.66	0.00

Smaller binding energy means more stable binding. RMSD: root mean square deviation. The RMSD was calculated by comparing the docked conformation with a reference structure (e.g., crystal structure)
